# Benefits of the multiplanar and volumetric analyses of pancreatic cancer using computed tomography

**DOI:** 10.1371/journal.pone.0240318

**Published:** 2020-10-07

**Authors:** Moon Hyung Choi, Seung Bae Yoon, Meiying Song, In Seok Lee, Tae Ho Hong, Myung Ah Lee, Eun Sun Jung

**Affiliations:** 1 Cancer Research Institute, College of Medicine, The Catholic University of Korea, Seoul, Korea; 2 Department of Radiology, College of Medicine, The Catholic University of Korea, Seoul, Korea; 3 Department of Internal Medicine, College of Medicine, The Catholic University of Korea, Seoul, Korea; 4 Department of Surgery, College of Medicine, The Catholic University of Korea, Seoul, Korea; 5 Department of Hospital Pathology, College of Medicine, The Catholic University of Korea, Seoul, Korea; MD Anderson Cancer Center, UNITED STATES

## Abstract

Although pancreatic cancer tumors are irregularly shaped in terms of their three-dimensional (3D) structure, when T staging by imaging results, generally only the axial plane is used to measure the largest tumor diameter. We investigated the size of pancreatic cancer tumors using multi-plane and 3D reconstructed computed tomography (CT) images and investigated their clinical usefulness. Patients who underwent surgery for pancreatic adenocarcinoma were included. We measured the largest diameter of each pancreatic tumor in the axial, coronal, and sagittal planes of CT images. In addition, maximal diameter and cancer volume were measured from 3D images that were constructed using a semi-automated software system. Final data were compared with pathologic examination and the effect of each value on prognosis was analyzed. A total of 183 patients were analyzed. The maximal diameters measured on the axial, coronal, and sagittal planes were 2.9 ± 1.1, 3.2 ± 0.9, and 3.2 ± 1.0 cm, respectively, which were significantly smaller than pathologic results (3.4 ± 1.4 cm, all *p*<0.05 by paired t-test). The longest diameter among them (3.4 ± 1.1 cm) was nearly similar to the pathologic diameter. Cancer volume measured on 3D images demonstrated a higher area under the receptor operating characteristic curve [0.714, (95% confidence interval: 0.640–0.788)] for predicting early death compared to any unidimensional CT diameters measured. The longest pancreatic tumor diameter measured on multiplanar CT images was most accurate when compared to its corresponding pathologic diameter. Tumor volume had a stronger correlation with overall survival than tumor diameter.

## Introduction

Although the incidence of pancreatic cancer has been increasing recently, the 5-year survival rate is still about 5% [1]. Surgical resection is the only curative option for pancreatic cancer treatment. Surgical techniques and perioperative management have improved over the past several decades, however, prognosis after surgery for pancreatic cancer remains poor [2–4]. In recent years, neoadjuvant therapy has been shown to improve resectability and survival in borderline resectable or localized pancreatic cancer [5–8]. It is necessary to identify the factors that can accurately assess prognosis before surgery and help to determine therapeutic strategies (i.e., upfront surgery versus neoadjuvant therapy).

In the new 8^th^ edition of the American Joint Commission on Cancer (AJCC) and the Union for International Cancer Control (UICC) system, size-based T staging was emphasized. In the new 8^th^ edition, T1 (≤2 cm), T2 (2–4 cm), and T3 (>4 cm) are classified only by the maximal diameter of the tumor [9]. Size-based T-staging is both simple and reproducible. It also has been shown to stratify the prognosis well in recent studies [10, 11].

Pathologic results are used for staging post-operatively, whereas image findings are used for staging at the time of treatment planning. According to the AJCC and UICC 8^th^ guidelines, it is recommended that T stage be assessed by measuring the largest tumor diameter in the axial plan using computed tomography (CT) or magnetic resonance images. However, a previous study reported that tumor diameter measured in the axial plane in CT images underestimated tumor size by about 20% and the association with prognosis and CT diameter was not significant [12]. Because pancreatic cancer tumors have a three-dimensional (3D) structure, it is likely that the axial diameter on CT is not the longest tumor diameter. Therefore, multiplanar and 3D analysis of pancreatic cancer tumors may be helpful for better predicting prognosis and in determining treatment policy.

In this study, we report the diameters of pancreatic cancer tumors in the axial, coronal, and sagittal planes that were obtained from preoperative CT images. We also evaluated tumor diameter and volume using 3D reconstructed CT images. We compared the diameters measured in the three anatomical planes with pathological results. In addition, we examined how these size factors correlated with overall survival after surgery.

## Materials and methods

### Patients and data collection

Patients newly diagnosed with pancreatic adenocarcinoma between 2009 and 2016 at Seoul St. Mary’s Hospital, Seoul, Korea were retrospectively analyzed. Patients diagnosed with pancreatic adenocarcinoma and who underwent surgery (i.e., either pancreaticoduodenectomy or distal pancreatectomy) were included in this study. Exclusion criteria for the study were (1) cases without initial CT performed at our institution, (2) patients who underwent palliative surgery, (3) patients who died or were lost to follow up within 2 months after surgery, and (4) pancreatic cancer arising from intraductal papillary mucinous neoplasms.

Preoperatively the following clinical data were collected: patient demographics, laboratory findings including carbohydrate antigen (CA) 19–9, and radiologic findings. Postoperatively, operative data, adjuvant chemotherapy, and follow-up data were also collected. The pathologic reports of all specimens in our study have been reviewed by a single pathologist (ESJ) with more than 20 years’ experience. Pathologic features included tumor stage, tumor size, tumor grade, and resection margin status. Resection margin involvement was defined as tumor cells in the margin or within 1 mm of the margin. None of our patients received neoadjuvant chemo- or radiation therapy. The Institutional Review Board of Seoul St. Mary’s Hospital approved this study (KC18RESI0496) and the informed consents were waived because of its retrospective design.

### Computed tomography-based image analysis

**Fig 1:**
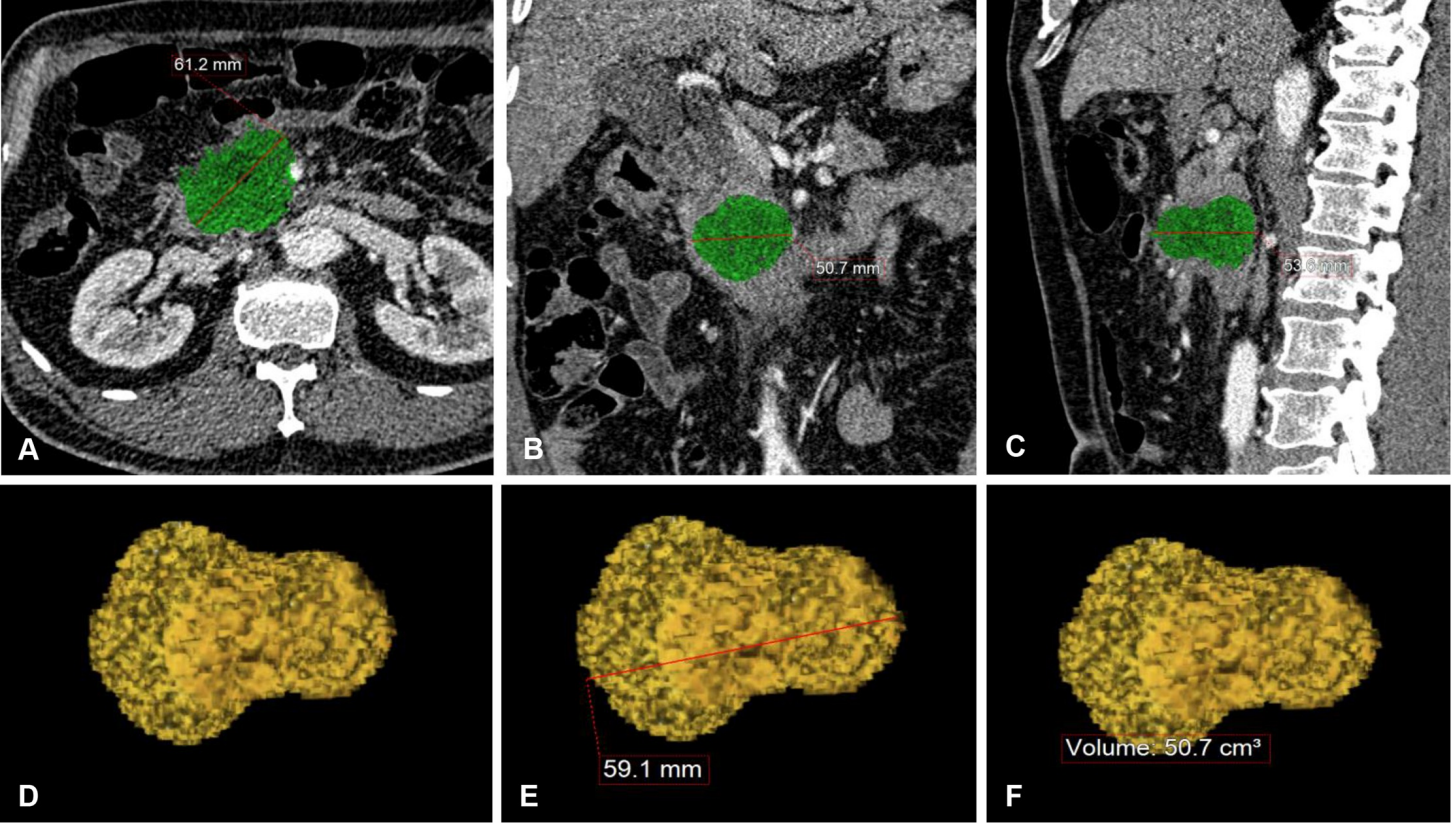
Measuring the diameter and volume of pancreatic cancer in multi-planes and three-dimensional (3D) computed tomography (CT) images. The maximal tumor diameters were measured in the axial (A), coronal (B), and sagittal (C) planes using CT images. A 3D CT image for each tumor was obtained through a semi-automated segmentation method (D), and maximal diameter on 3D images (E) and cancer volume (F) were measured.

Initial CT images before surgery were retrieved from a picture archiving and communication system for analysis. For CT-based image analysis, we used the same portal phase image for each tumor (120kVp, 180mAs) obtained with a fixed 75-second delay after contrast injection (iopromide; Ultravist, Bayer AG, Berlin, Germany). CT images were obtained with a 128- or 64-slice CT scanner (Somatom Definition/Somatom Definition AS+, Siemens Healthineers, Erlangen, Germany; Discovery CT750 HD, GE Healthcare, Chicago, IL) using a slice thickness of 3-mm with no gap. A radiologist (MH.C), blinded to patient information, performed image analysis. When the tumor margin was not clearly demarcated on the portal phase images, she referred to other CT images. Segmentation of the tumor on CT images was conducted using a workstation (TeraRecon Aquarius Workstation, TeraRecon Inc, San Mateo, CA, USA) with a semi-automated program that allowed the region-of-interest to grow based on similar image density. Then the radiologist trimmed the region-of-interest at the margin of the tumors slice by slice. She, then measured the maximal tumor diameters in the axial, coronal, and sagittal CT planes (Fig 1). Three-dimensional tumor images were reconstructed after complete image segmentation then tumor volume was automatically measured. After observing the 3D reconstructed tumor image, the maximal diameter was measured in the plane where the tumor was largest.

### Statistical analysis

Continuous data are presented as the mean ± SD or median with interquartile ranges (IQR), and categorical data are presented as quantity and proportion. Descriptive statistics were used to analyze the baseline characteristics of the study population. Comparisons of values between parameters were performed using paired *t*-tests. Overall survival was estimated using the Kaplan-Meier method, and the differences between groups were compared using the log-rank test. Receiver operating characteristic (ROC) curves predicting early death after surgery were constructed for various size parameters. The area under the curve (AUC) was calculated, and each AUC was compared by the Delong method. The maximal chi-square method was used to determine which value of tumor volume best segregated patients into good- and poor-prognosis after surgery. The impact of tumor volume and other parameters on overall survival was also examined using uni- and multivariable Cox proportional hazard models. Statistical analysis was performed using R 2.13.0 (R Development Core Team, Vienna, Austria) and MedCalc version 12.2.1 (MedCalc, Mariakerke, Belgium). Statistical significance was defined as p<0.05.

## Results

### Study population

A total of 237 patients underwent surgery for pancreatic adenocarcinoma during the study period. Of these, 21 patients had no preoperative CT performed at our institution, 9 patients underwent surgery for the purpose of palliative therapy, 7 patients died or were lost to follow up within 2 months after surgery, and 17 patients had tumors arising from intraductal papillary mucinous neoplasms. After excluding these 54 patients, the remaining 183 patients were analyzed. There were no missing values for all variables in the remaining 183 patients.

Study patient baseline characteristics (N = 183) are shown in Table 1. The mean age was 65.1 ± 9.4 years, and there were 99 (54.1%) males and 84 (45.1%) females. Among them, 157 (85.8%) patients received adjuvant chemotherapy for their cancer. Maximal tumor diameter according to pathologic results was 3.4 ± 1.4 cm and regional lymph node involvement was noted in 109 (59.6%) patients. According to the AJCC 8^th^ edition, the pathologic stage was classified as stage I in 56 (30.6%) patients, stage II in 95 (51.9%) patients, and stage III in 32 (17.5%) patients. Surgical margins were negative (R0) in 126 (69.8%) patients.

**Table 1:** Patient and tumor characteristics (N = 183).

Parameters	Data
**Patient Characteristics**	
Age, years	65.1 ± 9.4
Sex, male (%)	99 (54.1%)
CA 19–9, median (IQR), U/mL	106 (30–351)
Receiving adjuvant chemotherapy (%)	157 (85.8%)
**Tumor Characteristics**	
Primary tumor size, cm	3.4 ± 1.4
Regional lymph node positivity (%)	109 (59.6%)
Stage of tumor	
Stage I (%)	56 (30.6%)
Stage II (%)	95 (51.9%)
Stage III (%)	32 (17.5%)
Grade of tumor	
Well differentiated (%)	20 (10.9%)
Moderately differentiated (%)	147 (80.3%)
Poorly differentiated (%)	16 (8.7%)
Type of operation	
Pancreaticoduodenectomy (%)	133 (72.7%)
Distal pancreatectomy (%)	50 (27.3%)
Resection margin	
R0 resection (%)	126 (68.9%)
R1 resection (%)	57 (31.1%)

CA 19–9, carbohydrate antigen 19–9.

### Tumor diameters and volume

**Fig 2:**
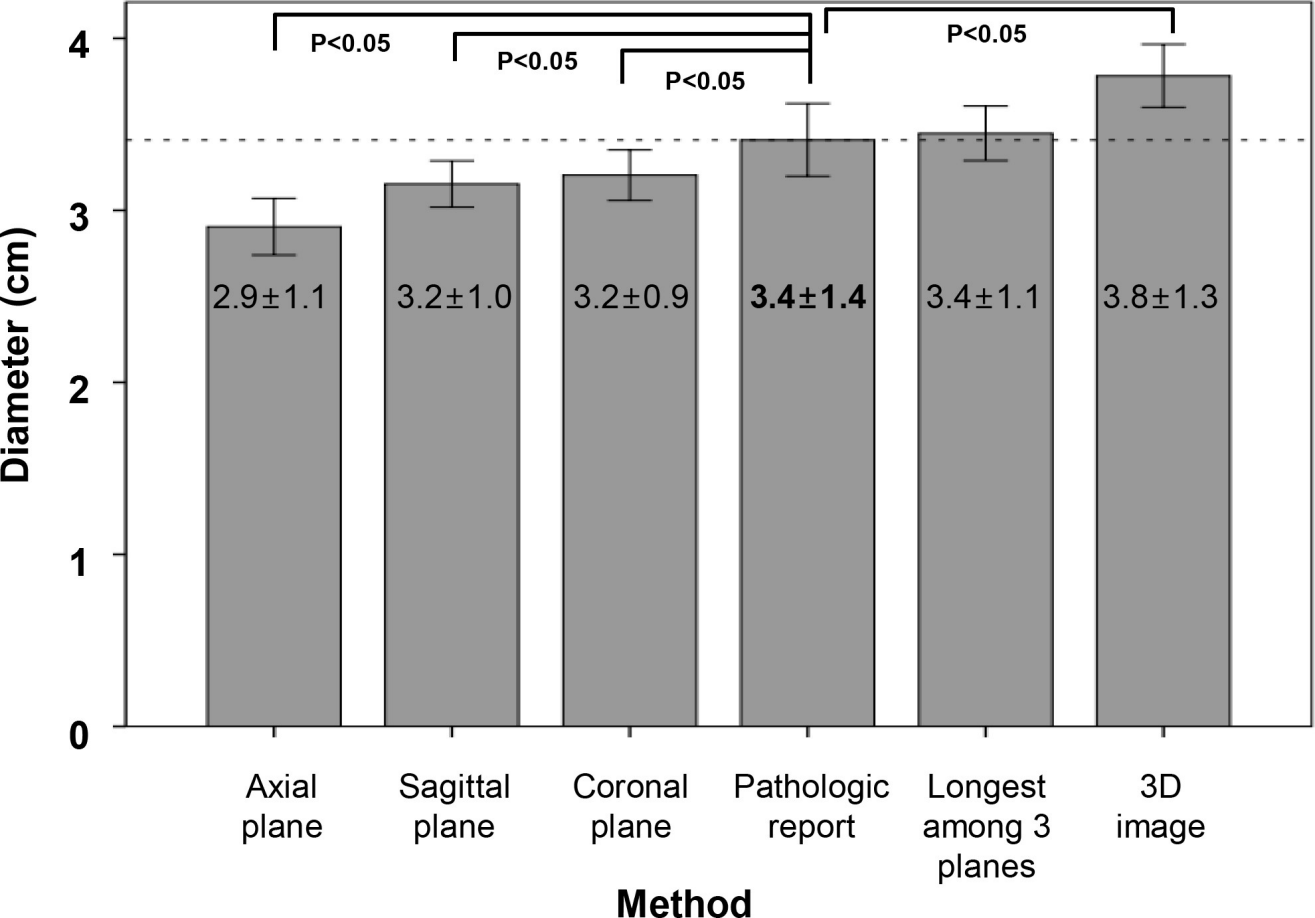
Comparison of pancreatic cancer diameters measured by various methods. Maximum tumor diameters measured in the axial, sagittal, and coronal CT planes were 2.9 ± 1.1, 3.2 ± 1.0, and 3.2 ± 0.9 cm, respectively.

Maximum tumor diameters measured in the axial, sagittal, and coronal CT planes were 2.9 ± 1.1, 3.2 ± 1.0, and 3.2 ± 0.9 cm, respectively (Fig 2). There were no significant differences among them. All the diameters measured on these three planes were significantly shorter than the maximal pathologic diameter (all *p*<0.05 by paired t-test). The longest diameter among the axial, sagittal, and coronal planes was 3.4 ± 1.1 cm and this was similar to the pathologic result, and was not significantly different (*p* = 0.642). The maximal diameter measured on 3D reconstructed images (3.8 ± 1.3 cm) was longer than the pathologic tumor diameter (*p*<0.05). Mean cancer volume in 3D images was measured as 12.5 ± 18.0 cm^3^.

### Survival analyses

**Fig 3:**
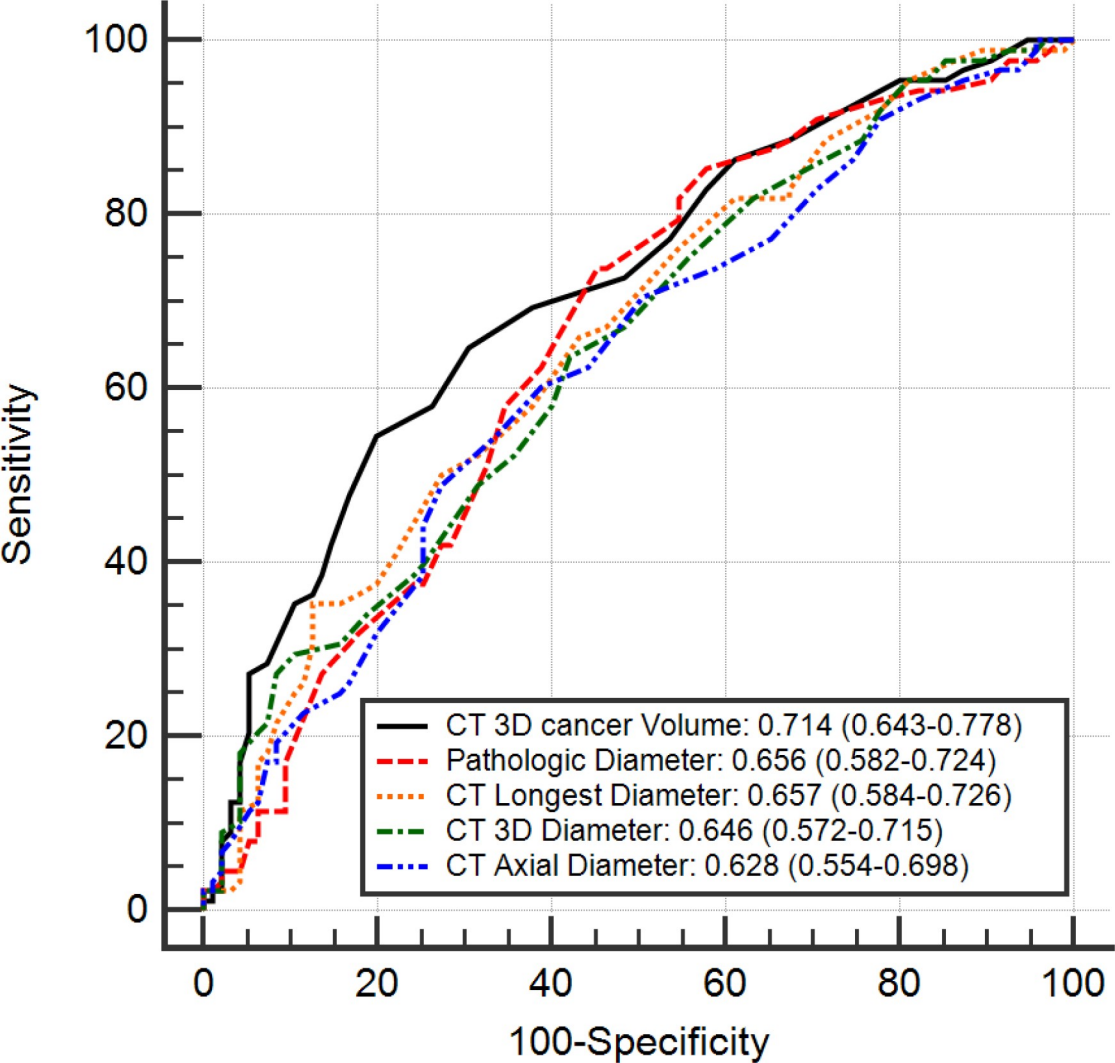
Receiver operating characteristic curves for different methods measuring pancreatic tumor size in predicting early death within 18 months. Cancer volume measured on 3D CT images demonstrated the highest area under curve (0.714; 95% confidence interval [CI], 0.643–0.778) among various size parameters.

The median overall survival after surgery for the entire cohort was 18.1 months. The cut-off value for early death was set at 18 months for clinical convenience. The AUCs for predicting early death were measured to compare various size parameters’ efficacy in predicting prognosis after surgery (Fig 3). Cancer volume measured on 3D CT images demonstrated the highest AUC (0.714; 95% confidence interval [CI], 0.643–0.778) among various size parameters. The AUC for tumor volume was significantly higher than those for the largest diameter among the three anatomical planes (0.657, 95% CI, 0.584–0.726), maximal 3D diameter (0.646; 95% CI, 0.572–0.715), and axial diameter (0.628; 95% CI, 0.554–0.698, Table 2). There was no significant difference between the AUC for tumor volume and pathologic diameter (0.656; 95% CI, 0.582–0.724). In addition, there were no significant differences between the AUCs for pathologic diameter and any imaging diameters.

**Table 2:** Pairwise comparison of P values for area under the curve in predicting early death after pancreatic cancer resection.

	CT 3D volume	Pathologic diameter	CT longest diameter among 3 planes	CT 3D diameter	CT axial diameter
CT 3D volume		0.161	0.027*	0.010*	0.009*
Pathologic diameter	0.161		0.974	0.810	0.583
CT longest diameter among 3 planes	0.027*	0.974		0.513	0.241
CT 3D diameter	0.010*	0.810	0.513		0.368
CT axial diameter	0.009*	0.583	0.241	0.368	

3D, three-dimensional.

**Fig 4:**
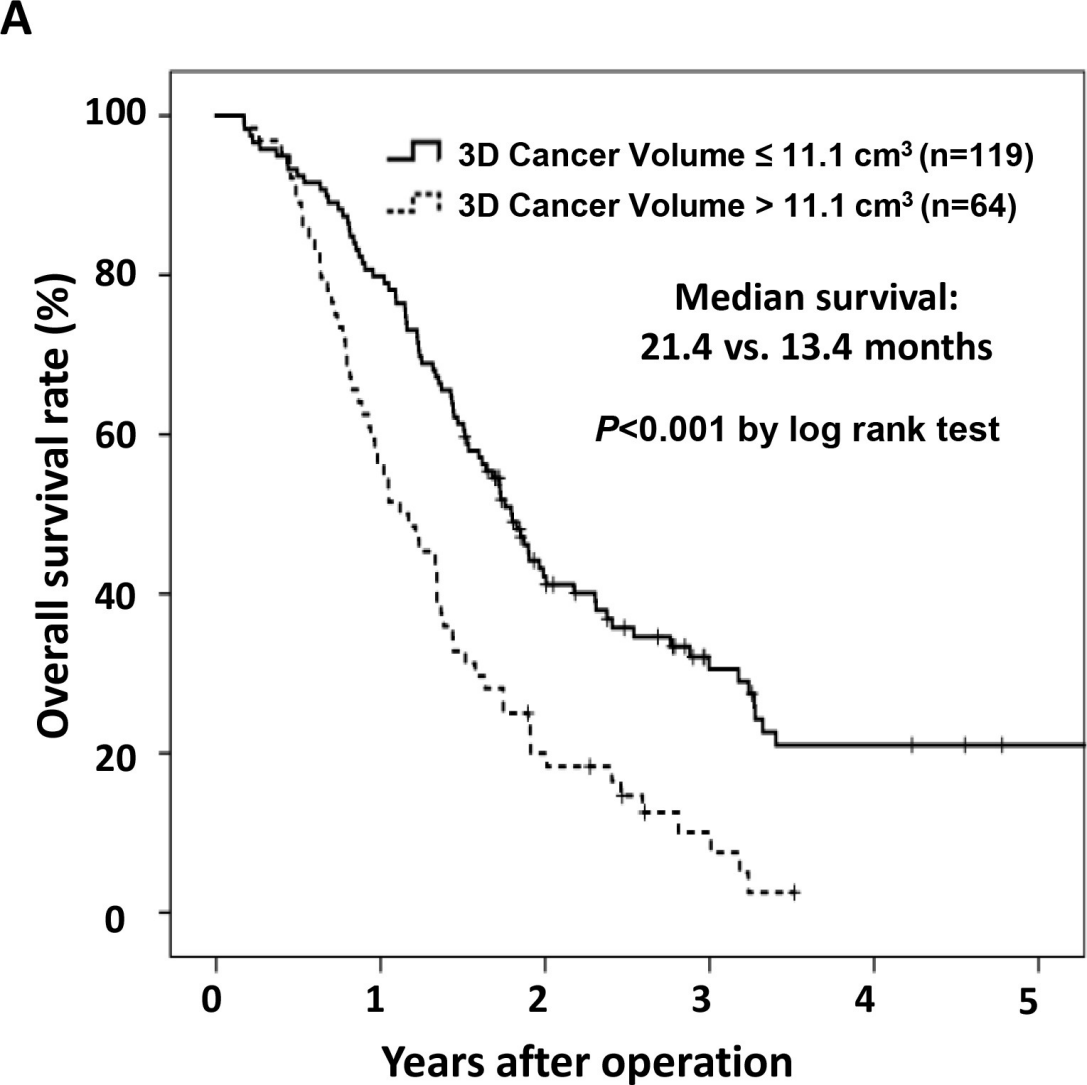
Overall survival curves after surgery according to the tumor volume cut-off level (11.1 cm^3^). The median survival of the small-volume group (21.4 months) was significantly longer than that of large-volume group (13.4 months, *p*<0.001).

The maximal chi-square method found that segregation of survival was best achieved by using a tumor volume of 11.1 cm^3^ as the cut-off value (Fig 4). By this criterion, 119 (65.0%) patients had a small tumor volume and 64 (35.0%) had a large tumor volume. The median survival of the small-volume group (21.4 months) was significantly longer than that of large-volume group (13.4 months, *p*<0.001). No significant differences were found in regional lymph node positivity (58.0% *vs*. 62.5%, *p* = 0.553) and R1 resection rate (28.6% *vs*. 35.9%, *p* = 0.305) between small-tumor and large-tumor volume groups. In addition, when ROC analyses were conducted, tumor volume could not predict the regional lymph node positivity (0.549; 95% CI, 0.461–0.637) and R1 resection rate (0.564; 95% CI, 0.475–0.653).

### Multivariable analyses

Uni- and multivariable Cox proportional hazard models for overall survival after resection of pancreatic cancer tumors are summarized in Table 3. Univariable Cox analysis showed that 3D tumor volume [hazard ratio (HR), 1.01; 95% CI, 1.01–1.02], higher CA 19–9 level than the median level (> 106 U/ml, HR, 1.50; 95% CI, 1.06–2.11), nodal metastasis (HR, 1.73; 95% CI, 1.22–2.44), R1 resection (HR, 1.62; 95% CI, 1.14–2.30), and adjuvant chemotherapy (HR, 0.59; 95% CI, 0.37–0.93) were significantly associated with overall survival. Multivariable analysis showed larger tumor volume measured on 3D CT images remained as a risk factor for poorer overall survival (HR, 1.02; 95% CI, 1.01–1.03).

**Table 3:** Cox proportional hazards models for overall survival after pancreatic cancer resection.

Factor	Univariable analysis		Multivariable analysis	
	HR (95% CI)	*p* value	HR (95% CI)	*p* value
Age	1.00 (0.98–1.02)	0.836	-	-
Male	1.25 (0.87–1.79)	0.225	-	-
**CT 3D Cancer volume (cm**^**3**^**)**	**1.01 (1.01–1.02)**	**0.001**	**1.02 (1.01–1.03)**	**<0.001**
Higher CA19-9 (> 106 U/ml, median level)	1.50 (1.06–2.11)	0.021	1.46 (1.37–2.10)	0.031
Lymph node metastasis	1.73 (1.22–2.44)	0.002	1.61 (1.11–2.33)	0.013
Poorly differentiated	1.59 (0.96–2.62)	0.071	-	-
R1 resection	1.62 (1.14–2.30)	0.007	1.62 (1.12–2.37)	0.011
Adjuvant chemotherapy	0.59 (0.37–0.93)	0.022	0.47 (0.29–0.75)	<0.002

3D, three-dimensional; CA 19–9, carbohydrate antigen 19–9.

## Discussion

In this study, we examined the diameters and volumes of pancreatic cancer tumors from multiplanar and 3D CT images and evaluated their accuracy compared to pathologic specimens and image measurement prognostic value. The longest tumor diameter measured among multiplane images showed the best correlation with the diameter obtained from pathologic specimen measurement. In addition, volumetric analysis of pancreatic cancer tumors was associated with better predictive outcomes than those obtained using the unidimensional analysis method.

There is an emphasis on primary tumor size for T-staging in the 8^th^ edition of the AJCC and UICC system for pancreatic cancer prognosis. Thus, accurate lesion size measurement on imaging findings (i.e., CT) has become important in preoperative settings. For pulmonary nodules, size measurement on multiplanar images that include the coronal and sagittal planes has been recommend by an expert group [13]. However, according to the AJCC and UICC guidelines for pancreatic cancer, T-stage is assessed using the longest tumor diameter in the axial plane. In addition, there have been no studies that analyzed the diameter of pancreatic cancer tumors using a multiplanar approach. According to our study, the diameters measured in the axial, coronal, and sagittal planes were all shorter than the diameters obtained by pathologic measurement. The longest diameter among the three anatomical planes was not different from the pathological diameter and was more accurate than the axial diameter. These results were consistent with the results of recent studies conducted in lung cancer patients [14–16]. Pancreatic cancer tumors tend to grow unevenly in different directions, therefore, we believe that multiplanar measurements are necessary for preoperative clinical staging to accurately assess primary lesion size.

Theoretically, we expected that the maximal diameter measured on 3D CT images would be the closest to the pathologic diameter. However, the diameter on 3D images tended to overestimate tumor size compared with pathologic size, as also shown in previous studies on lung cancer [17–19]. It is assumed that the longest diameter of the 3D reconstructed tumor might be exaggerated depending on the angle between the observer and the tumor. It is also possible that the pathologic resected specimen might have shrunk or been deformed during tissue processing.

Although unidimensional measurements have been the mainstay of size-based imaging assessment, volumetric analysis has potential clinical advantages. A recent study showed that volumetric analysis of pancreatic tumors could be useful in assessing response evaluation in palliative settings [20]. For the first time, our study shows that preoperative volume analysis of pancreatic tumors was more associated with postoperative survival compared to unidimensional image analysis. An advantage of volumetric analysis may be that this method represents the full extent of a lesion even it is irregular.

In our institution, neoadjuvant chemo-or chemoradiotherapy was not considered in pancreatic cancer patients during the study period. Currently, however, neoadjuvant treatment has become a mainstay for the treatment of borderline resectable or locally advanced pancreatic cancer [21]. Furthermore, a recent study suggested that neoadjuvant therapy for patients with resectable pancreatic cancer could provide outcome improvement in terms of median overall survival [22]. Therefore, it is suggested that clinicians strongly consider the use of neoadjuvant treatment even in resectable appearing T2 or T3 tumors. In our study, the median overall survival in the large-volume group (>11.1 cm^3^) undergoing upfront surgery was 13.4 months, which was similar to the known median survival of locally advanced cancer patients (between 13 and 17 months) receiving chemotherapy without surgery [23, 24]. Therefore, in a situation where neoadjuvant treatment is considered for management of pancreatic cancer, 3D cancer volume can be used as a biomarker. To do that, validation in a large number of patients will be required to verify the cut-off value we have found.

Further, the multivariable analysis in our study showed that prognosis after pancreatic tumor resection depended on various factors including regional lymph node metastasis, resection curability, and adjuvant chemotherapy. However, all of these factors mentioned above can be only determined after surgery. In real clinical settings, only CA19-9 and tumor volume can be candidate prognostic markers to help to make preoperative clinical decisions. It is expected that the predictive model developed by combining these two factors will help guide the clinical practice of pancreatic tumor resection and treatment.

Despite advances in 3D visualization platforms that enable volumetric analysis, this process requires more labor and time than unidimensional measurement. In addition, it is difficult for all institutions to acquire an advanced visualization software program and trained clinicians that allow for reliable 3D analyses. On the other hand, because multiplanar analysis has been widely used, multiplanar and/or 3D analyses can be performed appropriately, depending on the capability and circumstances of each institution.

There were some limitations to our study. First, our study was limited by its retrospective nature. Second, this study was conducted in a single institution and included only East Asian pancreatic cancer patients (Korean). Third, we could not find any radiologic characteristics of tumor predicting lymph node metastasis or R1 resection which would be more important in the real clinical setting. Finally, this research was conducted only in patients undergoing upfront surgery, so it is difficult to apply our findings directly to neoadjuvant treatment patients. A future prospective validation study of the setting of neoadjuvant chemotherapy will be needed to confirm and elaborate on our findings.

In conclusion, multiplanar analysis using CT allowed more accurate measurement of the size of pancreatic tumors before surgery. Volume analysis was more useful in predicting overall survival after surgery than unidimensional analysis.
